# Sex-Specific Differences in Primary CNS Lymphoma

**DOI:** 10.3390/cancers12061593

**Published:** 2020-06-16

**Authors:** Thomas Roetzer, Julia Furtner, Johanna Gesperger, Lukas Seebrecht, Dave Bandke, Martina Brada, Tanisa Brandner-Kokalj, Astrid Grams, Johannes Haybaeck, Melitta Kitzwoegerer, Stefan L. Leber, Franz Marhold, Patrizia Moser, Camillo Sherif, Johannes Trenkler, Julia Unterluggauer, Serge Weis, Franz Wuertz, Johannes A. Hainfellner, Georg Langs, Karl-Heinz Nenning, Adelheid Woehrer

**Affiliations:** 1 Division of Neuropathology & Neurochemistry, Department of Neurology, Medical University of Vienna, 1090 Vienna, Austria; thomas.roetzer@meduniwien.ac.at (T.R.); johanna.gesperger@meduniwien.ac.at (J.G.); lukas.seebrecht@meduniwien.ac.at (L.S.); johannes.hainfellner@meduniwien.ac.at (J.A.H.); adelheid.woehrer@meduniwien.ac.at (A.W.); 2 Department of Biomedical Imaging and Image-guided Therapy, Division of Neuroradiology and Musculoskeletal Radiology, Medical University of Vienna, 1090 Vienna, Austria; julia.furtner@meduniwien.ac.at; 3 Center for Medical Physics and Biomedical Engineering, Medical University of Vienna, 1090 Vienna, Austria; 4 Division of Neuropathology, NeuromedCampus, Kepler University Hospital, Johannes Kepler University, 4040 Linz, Austria; Dave.Bandke@kepleruniklinikum.at (D.B.); serge.weis@kepleruniklinikum.at (S.W.); 5 Department of Pathology, Krankenanstalt Rudolfstiftung, 1030 Vienna, Austria; Martina.Brada@wienkav.at; 6 Institute of Pathology, State Hospital Klagenfurt, 9020 Klagenfurt, Austria; Tanisa.Brandner-Kokalj@kabeg.at (T.B.-K.); Franz.Wuertz@kabeg.at (F.W.); 7 Department of Neuroradiology, Medical University of Innsbruck, 6020 Innsbruck, Austria; astrid.grams@i-med.ac.at; 8 Diagnostic and Research Institute of Pathology, Medical University of Graz, 8036 Graz, Austria; Johannes.Haybaeck@i-med.ac.at (J.H.); j.unterluggauer@gmx.at (J.U.); 9 Department of Pathology, Neuropathology and Molecular Pathology, Medical University of Innsbruck, 6020 Innsbruck, Austria; 10 Department of Pathology, University Hospital St. Poelten, Karl Landsteiner University of Health Sciences, 3100 St. Poelten, Austria; Melitta.Kitzwoegerer@stpoelten.lknoe.at; 11 Division of Neuroradiology, Vascular and Interventional Radiology, Department of Radiology, Medical University of Graz, 8036 Graz, Austria; stefan.leber@medunigraz.at; 12 Department of Neurosurgery, University Hospital St. Poelten, Karl Landsteiner University of Health Sciences, 3100 St. Poelten, Austria; Franz.Marhold@stpoelten.lknoe.at; 13 Department of Pathology, Medical University of Innsbruck, 6020 Innsbruck, Austria; patrizia.moser@tirol-kliniken.at; 14 Department of Neurosurgery, Krankenanstalt Rudolfstiftung, 1030 Vienna, Austria; camillo.sherif@gmail.com; 15 Institute of Neuroradiology, NeuromedCampus, Kepler University Hospital, Johannes Kepler University of Linz, 4020 Linz, Austria; johannes.trenkler@kepleruniklinikum.at; 16 Computational Imaging Research Lab, Department of Biomedical Imaging and Image-guided Therapy, Medical University of Vienna, 1090 Vienna, Austria; georg.langs@meduniwien.ac.at

**Keywords:** PCNSL, DLBCL, sex-specific analyses, multimodal data, microenvironment

## Abstract

Sex-specific differences have been increasingly recognized in many human diseases including brain cancer, namely glioblastoma. Primary CNS lymphoma (PCNSL) is an exceedingly rare type of brain cancer that tends to have a higher incidence and worse outcomes in male patients. Yet, relatively little is known about the reasons that contribute to these observed sex-specific differences. Using a population-representative cohort of patients with PCNSL with dense magnetic resonance (MR) imaging and digital pathology annotation (*n* = 74), we performed sex-specific cluster and survival analyses to explore possible associations. We found three prognostically relevant clusters for females and two for males, characterized by differences in (i) patient demographics, (ii) tumor-associated immune response, and (iii) MR imaging phenotypes. Upon a multivariable analysis, an enhanced FoxP3+ lymphocyte-driven immune response was associated with a shorter overall survival particularly in female patients (HR 1.65, *p* = 0.035), while an increased extent of contrast enhancement emerged as an adverse predictor of outcomes in male patients (HR 1.05, *p* < 0.01). In conclusion, we found divergent prognostic constellations between female and male patients with PCNSL that suggest differential roles of tumor-associated immune response and MR imaging phenotypes. Our results further underline the importance of continued sex-specific analyses in the field of brain cancer.

## 1. Introduction

Sex-specific differences play an important yet probably not fully appreciated role in the majority of human diseases. They pertain to disease incidence and outcome and the underlying pathogenetic and/or molecular characteristics and provide a starting point for treatments tailored to male and female patients, separately [1,2]. For instance, sex-specific differences have been particularly well studied in cardiovascular diseases, where women show the increased signaling of protective hormonal pathways, such as the estrogen-induced modulation of the renin-angiotensin-aldosterone system, an advantage that is typically lost after menopause or in the presence of comorbidities such as obesity [3,4].

The importance of sex-specific analyses in cancer and particularly brain cancer has recently been highlighted by a seminal study on glioblastoma, the most common malignant brain tumor in adults that tends to be more common and aggressive in males. By splitting their molecular data in male and female patients, applying specific bioinformatic methods, and introducing sex-specific stratification in functional validation experiments, the authors were able to identify previously unrecognized tumor molecular, magnetic resonance (MR) imaging, and pharmacokinetic factors that explain the observed survival differences [5]. One particularly relevant aspect that has been recognized from that and similar analyses relates to the opposing effect sizes in females and males, which might cancel each other when using conventional joint analyses [5,6].

Primary CNS lymphoma (PCNSL) is a rare type of extra-nodal non-Hodgkin lymphoma that primarily affects the brain and its coverings. It is another example of a brain cancer where sex-specific differences have been implicated based on a higher disease incidence and worse outcomes in male patients [7,8]. While treatment is less standardized in PCNSL as compared to systemic lymphoma or primary brain tumors [9,10], the current guidelines agree upon high-dose methotrexate (HD-MTX) as an important pillar. Over the last few years, immune-therapeutic approaches have been increasingly recognized, including anti-CD20 rituximab [11,12,13], the immune-modulatory drugs lenalidomide/pomalidomide [14,15,16], a checkpoint blockade using anti-PD1 antibodies [17], or the transfer of chimeric antigen receptor (CAR) T cells [18,19].

The risk factors for poor outcomes include impaired immune status, increased patient age, poor clinical performance, and the non-receipt or failure of first-line chemotherapy [20,21,22,23,24,25]. In addition, a negative prognostic role has been postulated for the involvement of deep brain structures [24,25,26]. In contrast, analyses of tumor-infiltrating lymphocytes (TILs) and tumor-associated macrophages (TAMs) have yielded controversial or inconclusive results [27,28,29,30,31,32] and were found to differ between CNS and non-CNS lymphoma [33,34].

In contrast to the long-implicated differences in disease occurrence and patient outcome between female and male patients, no comprehensive sex-specific analysis has been conducted in PCNSL to date. Here, we set out to first explore sex-specific differences in a large, population-representative cohort using dense clinical, MR imaging, and digital pathology annotations. 

## 2. Materials and Methods

### 2.1. Patient Inclusion and Clinical Data

All the patients in this retrospective study were identified and included using a nation-wide disease registry—i.e., the Austrian Brain Tumor Registry (ABTR), which uses multiple sources for case reporting [35]. We searched the registry for patients with a first diagnosis of PCNSL between January 2005 and December 2010 who were over 18 years of age at the time of diagnosis (*n* = 189). The last follow-up was in September 2019. We collected clinical data from medical records across all the cooperating centers, including information on age, sex, first-line treatment (receipt of chemotherapy including the use of MTX, radiotherapy, rituximab), immune status, performance status (according to the Eastern Cooperative Oncology Group (ECOG) scale), last follow-up, and the survival status at the end of the follow-up. A detailed pattern-of-care analysis has recently been reported elsewhere [10]. Briefly, survival has particularly improved among younger patients and, while therapeutic strategies remained heterogeneous, MTX-based poly-chemotherapy and rituximab immunotherapy have been increasingly used in neuro-oncological practice. Of note, impaired immune status was caused by HIV-infection in only one patient. We now extend this dataset by adding MR imaging and digital pathology annotations (see section below). For a subset of 74 patients, both MR imaging data and sufficient formalin-fixed paraffin-embedded tissues were available. 

All the investigations were carried out following the rules of the Declaration of Helsinki and approved by the ethics committee of the Medical University of Vienna (EK# 1514/2018).

### 2.2. MR Imaging Data

The MR imaging dataset comprised T1-weighted images pre and post contrast enhancement (CE), fluid-attenuated inversion recovery (FLAIR), and T2-weighted image sequences. For each patient, the images were co-registered to the T1-CE acquisition and manually segmented by an expert radiologist into three tumor categories—i.e., enhancing tumor, necrosis, and edema/non-enhancing tumor. Based on that segmentation, we quantified the volumes of the respective tumor categories in cm^3^. Furthermore, we quantified the tumor location by determining the percentage of the main tumor mass lying in each of the following regions: frontal, parietal, temporal, occipital, and deep brain regions (comprising the corpus callosum, periventricular regions, basal ganglia, thalamus, brainstem, and cerebellum). For this purpose, an atlas was constructed by merging existing specialized atlases in the Montreal Neurological Institute (MNI) space for the lobes [36] and deep brain structures [37,38]. This atlas was warped to patient-specific images using Advanced Normalization Tools (ANTs) non-linear registration. We then automatically quantified the normalized location of the tumor by matching the patient-specific tumor segmentations to atlas regions. The resulting patient-specific mappings were manually inspected and carefully checked for registration errors. Furthermore, we evaluated the number of individual tumor foci (i.e., unifocal versus multifocal disease) by quantifying the number of groups of interconnected enhancing tumor voxels. 

### 2.3. Digital Pathology Data

We obtained formalin-fixed paraffin-embedded material from all 74 patients. A board-certified neuropathologist (A.W.) reviewed the H&E stained sections of all cases to confirm the diagnosis of a diffuse large B-cell lymphoma (DLBCL) and to identify representative tumor regions, which were subsequently punched and assembled into tissue microarrays (TMAs). We cut the TMAs at a thickness of 3 μm and immunohistochemically stained them for CD3 (Thermo Scientific, 1:200, pH6, Waltham, United States), CD45ro (DakoCytomation, 1:500, pH6, Glostrup, Denmark), and CD68 (DakoCytomation, 1:5000, pH6, Glostrup, Denmark) using a Dako autostainer system after antigen retrieval at 95 °C for 20 min at a given pH and antibody incubation for 30 min at room temperature. In addition, we stained FoxP3 (BioLegend, 1:25, San Diego, CA, United States) on a Ventana BenchMark automated staining system. We used diaminobenzidine (DAB) for staining and hematoxylin for counterstaining. We then scanned the stained slides using a Hamamatsu NanoZoomer 2.0 HT slide scanner using NDP.view2 image viewing software. We manually exported each TMA tissue core as a tiff-image in 10× magnification for further computational processing.

We used a custom MATLAB script (MATLAB, R2017b, MathWorks) to count the percentage of positively stained cells in each tissue core using a similar approach, as previously published [39,40]. In summary, we deconvoluted the hematoxylin (blue) and DAB (brown) color channels [41] and analyzed them separately. The hematoxylin or DAB-stained cells were locally and globally thresholded in an automatic way using Phansalkar’s [42] and Otsu’s methods [43]. Closely adjacent cells were split using a watershed algorithm [44]. To eliminate positively stained background pixels, only pixels with at least two positively stained neighbors were kept. Finally, the ratio of DAB+ to hematoxylin+ cells was calculated.

### 2.4. Cluster Analysis

We implemented clustering algorithms in python 3.7.4 using scikit-learn [45], matplotlib [46], NumPy [47], and SciPy [48]. After the data exploration, we imputed single missing data points using an iterative random forest imputer [49]. That is, five patients had missing clinical performance status, and two, one, and one patient(s) had missing CD3, CD45ro, and FoxP3 scores, respectively, due to single tissue cores floating off during the histological processing. To explore clusters at the time of diagnosis that were not biased by subsequent therapeutic parameters (that have a strong prognostic impact), we defined patient clusters using the following numerical/ordinal variables—deep location, MR imaging-based tumor volumetrics, number of foci, age, performance, and tumor-associated TIL/TAM scores—separately for female, male, and all patients. Except for deep location, which has previously been implicated as prognostically relevant, we excluded other location variables, since their values were found to be highly interdependent and therefore would dominate a cluster analysis. After z-scoring, we fitted an agglomerative clustering model with Euclidean distance and Ward-linkage to the respective patient cohort. To identify cluster-specific features, we compared the numerical and ordinal features with ANOVAs and Kruskal–Wallis H-tests, respectively.

### 2.5. Survival Analysis

To ensure a homogenous patient cohort, only patients with an intact immune status and receipt of first-line chemotherapy were included in the survival analysis (*n* = 56). We performed a Kaplan–Meier survival analysis for different patient subcohorts and clusters using the R survminer package [50]. We used log rank-tests to determine the *p*-values. For each numerical/ordinal variable, we determined the ideal cut-off in R (v. 3.6.1) using a recursive partitioning analysis with the rpart-package [51] for female, male, and all patients, separately. We selected all the simple variables (not complex cluster memberships) associated with the overall survival (OS) upon univariable analysis (*p* < 0.1) in any sub-cohort for multivariable Cox modeling using backwards elimination in R [52], and visualized the results with the survminer package.

### 2.6. Assessment of Intratumoral Heterogeneity

For 26 patients, multiple—i.e., 2–3 TMA—tissue cores from different regions of the same tumor biopsy were evaluated (multisector samples). For each patient, we correlated the immune scores across the multisector samples and calculated Pearson’s R and the corresponding *p*-values. Furthermore, we assessed the intratumoral distribution of CD3+ lymphocytes for one of the rare large tumor resections and visualized the pixel-wise percentages as a spatial heatmap. Therefore, we manually excluded artificially damaged regions and staining artifacts from further analysis. Then, we extracted the centroids of DAB- and hematoxylin-stained cells from the custom MATLAB script (see digital pathology data). In order to calculate the pixel-wise percentages of CD3+-stained cells—i.e., the ratio of the pixel-wise densities of DAB+ to hematoxylin+ cells—we used a Gaussian filter with a standard deviation of 204 pixels (=186 μm, corresponding to a Gaussian distribution with 99% of data points within a circle of 1 mm^2^).

### 2.7. Data and Code Availability

The MATLAB and python-code is available as a github repository (REF). The data is available via zenodo (REF). 

## 3. Results

### 3.1. General Characterization of the Patient Cohort

**Figure 1:**
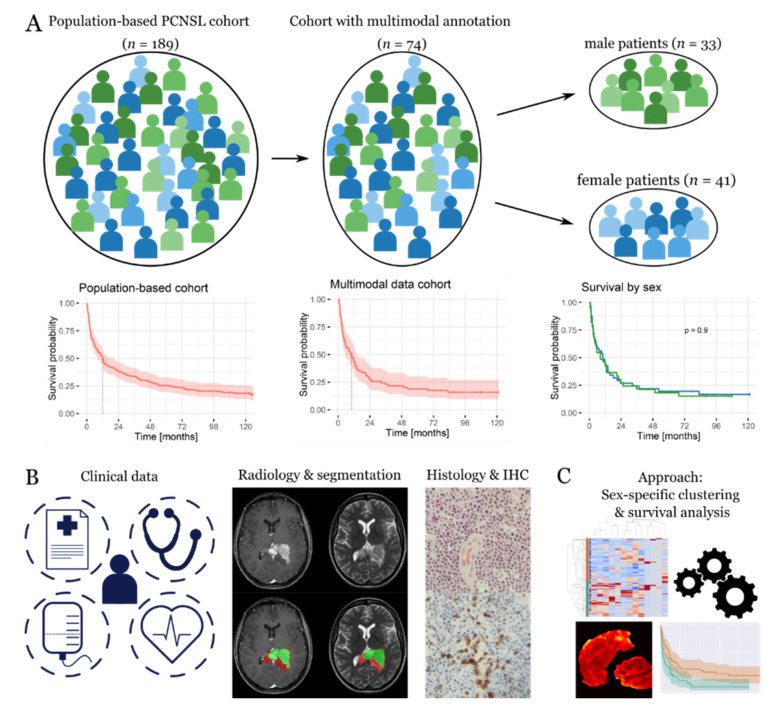
Population-representative cohort with dense phenotypic annotation. (**A**) Starting from a population-based cohort (*n* = 189), we retrieved dense phenotypic annotation for a sub-cohort of patients (*n* = 74) representative of the underlying population in terms of outcome. The dataset was split into female (*n* = 41) and male (*n* = 33) patients for a further sex-specific analysis. (**B**) The latter was based on clinical and therapeutic data; magnetic resonance imaging data, including tumor segmentations; and digital pathology data, including immune scores. magnification: ×20. (**C**) The data were integrated using cluster and survival analyses in a sex-specific manner.

The PCNSL cohort with a dense phenotypic annotation comprised 41 females and 33 males and was representative of the underlying patient population in terms of the sex composition and outcome (Figure 1A–C). The same was true for the patient age, performance status, immune status, and choice of treatment modality. An overview of the basic descriptors of all clinical, MR imaging, and digital pathology variables is given in Table 1. We found single differences between the female and male patients, including a higher prevalence of rituximab treatment among females (*p* = 0.02) as well as a higher occurrence of multifocal disease in males (*p* < 0.01). In contrast, no statistically significant differences were observed among other variables such as age, MTX-based therapy, MR imaging-based tumor volumetrics, and tumor-associated immune response.

### 3.2. Prognostic Constellations Differ between Females and Males

**Figure 2:**
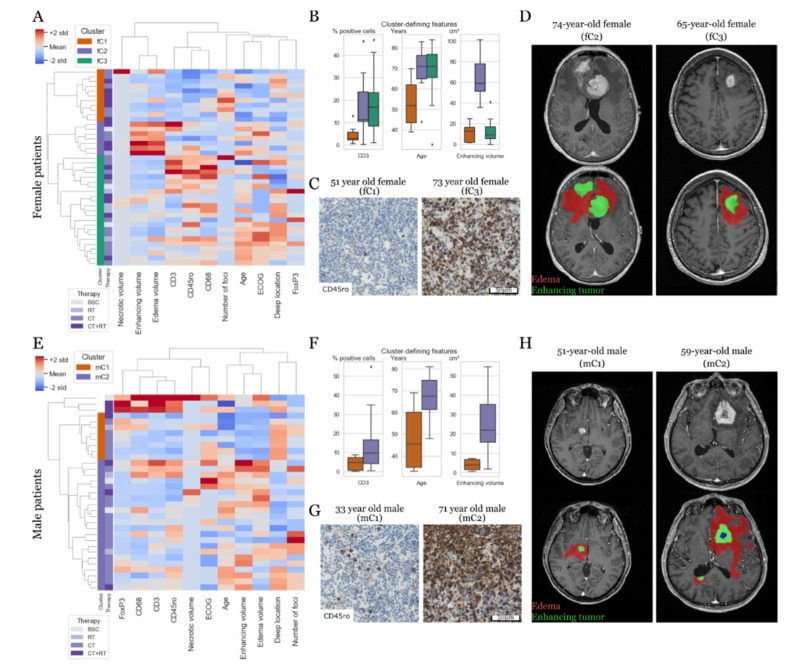
Cluster analysis (Euclidean distance, Ward-linked) based on numerical and ordinal variables. (**A**–**D**) Female patients. (**E**–**H**) Male patients. (**A**) Cluster analysis of the female dataset results in three clusters that significantly differ in the combination of features, three of which are depicted in (**B**), such as immune response (CD3+ TILs), age, and contrast enhancement (all *p* < 0.01, ANOVA). (**C**) Comparison of the CD45ro+ immune response of two representative female patients. (**D**) Comparison of the MR imaging features of two representative female patients. (**E**) Cluster analysis of male patients yields two main clusters. (**F**) Examples of cluster-defining features include immune response (CD3+ TILs), age, and contrast enhancement (all *p* < 0.01, ANOVA). (**G**) Comparison of the CD45ro+ immune response of two representative male patients. (**H**) Comparison of the MR imaging features of two representative male patients.

When evaluating sets of numerical and ordinal variables using a hierarchical cluster analysis, we found three main clusters in female patients (Figure 2A–D). The first cluster (fC1, 27%) was composed of younger patients (median age 53.5 years) with good clinical performance (ECOG ≤1), small lesions with limited contrast-enhancement and edema upon MR imaging, and little tumor-associated immune response. The second cluster (fC2, 17%) featured patients with significantly enlarged enhancing tumor volumes (>30 cm^2^) and edema (>100 cm^2^) upon MR imaging. The third cluster (fC3, 56%) was dominated by the increased anti-tumor immune responses of CD3+; CD45ro+; and FoxP3+ TILs, CD68+ TAMs, as well as tumors of deep location. Both older age and worse clinical performance were equally distributed among clusters fC2 and fC3. 

In males, two major clusters emerged (Figure 2E–H) with single cases that contributed to a minor third cluster that was driven by enhanced immune response. However, due to its small sample size (*n* = 3), we excluded it from further analyses. While the first cluster (mC1, 24 %) featured mainly younger patients (median age 47.9 years) with lesions in deep locations (typically > 60 % of the tumor mass), the second cluster (mC2, 67%) included patients with increased MR imaging-based enhancing tumor volume (typically > 10 cm^3^) and edema (typically > 50 cm^3^) in addition to an increased TIL/TAM immune response. We provide more detailed information on the cluster-defining variables in Table S1. Supplementary clustering results for the combined female/male patient cohort are shown in Figure S1.

### 3.3. Tumor-Associated Immune Response and MR Phenotypes Show Differential Prognostic Impact

**Figure 3:**
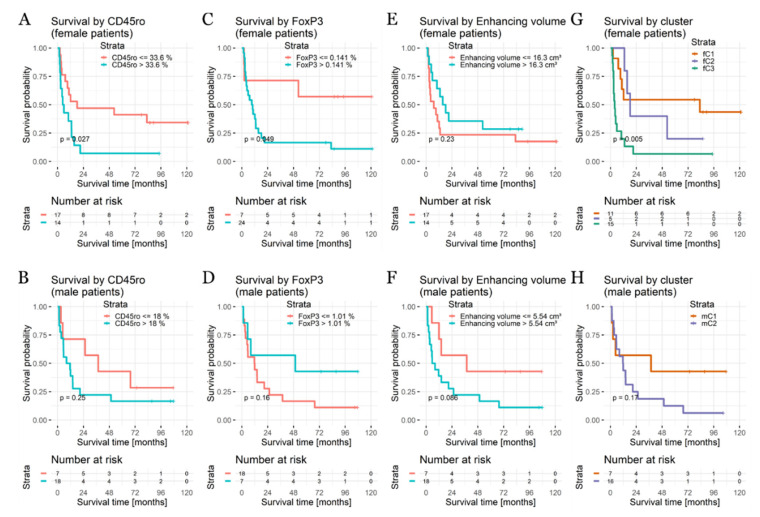
Selected Kaplan–Meier survival plots for variables with differential prognostic associations in female (upper row) and male patients (lower row). (**A**) In female patients, CD45ro+ TIL were associated with a worse survival (mOS 5.3 vs. 18.2 months, *p* = 0.027). (**B**) In contrast, there is no significant association in male patients (mOS 37.7 vs. 9.95 months, *p* = 0.25). (**C**) In female patients, FoxP3+ TILs are associated with worse survival (mOS 9.6 months vs. mOS not reached, *p* = 0.049), whereas in males (**D**) there is no association (mOS 49.6 vs. 11.8 months, *p* = 0.16). (**E**) While increased enhancing tumor volume does not affect survival in females (mOS 16.8 vs. 7.1 months, *p* = 0.23), male patients (**F**) with an increased contrast enhancement show a non-significant tendency towards worse outcomes (6.92 vs. 37.7 months, *p* = 0.09). (**G**) Cluster membership was associated with survival in female patients (mOS 3.9 vs. 18.2 vs. 82.9 months, *p* = 0.005, (**H**) but not in male patients (37.7 vs. 11.8 months, *p* = 0.17).

In the entire patient cohort, the median overall survival was 10.3 months in female patients (range, 1 day to 171 months) and 8.3 months in male patients (range, 6 days to 171 months), which was not significantly different (*p* = 0.97). For the subset of patients who had an intact immune status and received first-line chemotherapy (*n* = 56), the median overall survival was 10.5 months for females and 11.9 months for males (*p* = 0.98). Selected Kaplan–Meier plots are displayed in Figure 3, and complete results including the recursive partitioning analysis-obtained cut-offs for each variable per sex are given in Table S2.

Upon the univariable analysis, age and clinical performance were significant predictors of survival in females and males (all *p* < 0.02). In addition, tumor-associated immune response (CD3, *p* = 0.04; CD45ro, *p* = 0.03; FoxP3, *p* = 0.05)) and frontal tumor location (*p* = 0.01) were associated with longer overall survival (OS) in female patients. In males, there were no additional statistically significant predictors, even though enhancing the tumor volume (*p* = 0.09) and right-hemispheric tumor location (*p* = 0.07) showed non-significant tendencies towards shorter survival. Upon a multivariable analysis, advanced age and FoxP3-driven immune response remained significant adverse factors in females. In contrast, worse clinical performance, increased enhancing tumor volume, and low FoxP3 immune response were associated with a shorter OS in male patients (see Table 2). 

When looking at the prognostic relevance of sex-specific clusters, in females the immune responsive cluster fC3 showed the worst overall survival as compared with the MR phenotype-based “large tumor” cluster fC2, with intermediate survival and the young age cluster fC1 with the best survival (3.9 vs. 18.2 vs. 82.9 months, *p* = 0.005; Figure 3). In males, there was a non-significant trend toward a delayed survival benefit of cluster mC1, “young patients with tumors in deep location” (37.7 vs. 11.8 months, *p* = 0.17). 

### 3.4. Intratumoral Heterogeneity of Immune Response

**Figure 4:**
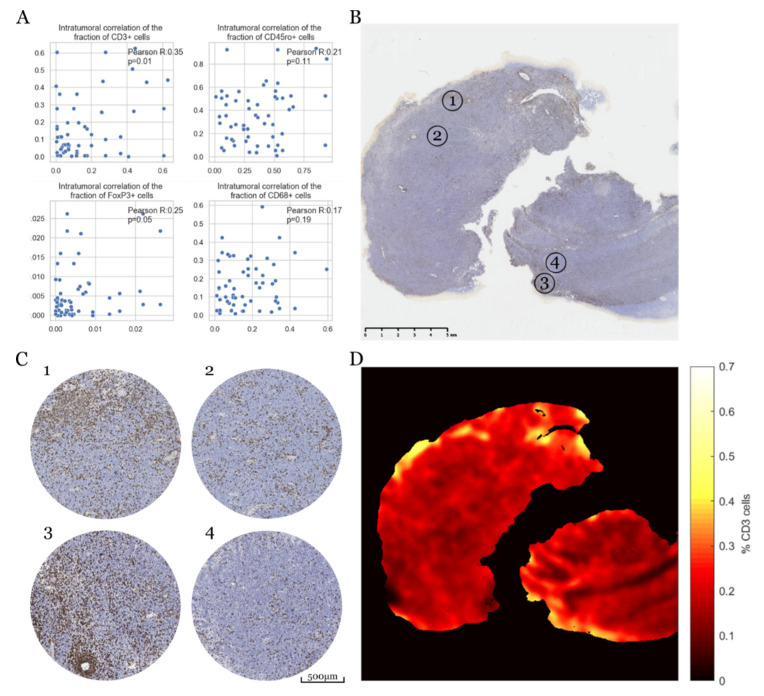
Spatially heterogeneous immune response in Primary CNS lymphoma (PCNSL). (**A**) Correlation of intratumoral TIL/TAM counts across multi-sector regions (*n* = 26). (**B**) CD3 TIL staining of a large PCNSL specimen. (**C**) Virtual tissue cores randomly sampled from four sectors annotated in (**B**) demonstrate significant heterogeneity between immune hot and cold spots. (**D**) Heatmap of the CD3+ cell density with immune hot and cold spots across the whole slide scan.

As an intense anti-tumor immune response emerged as a prognostically relevant factor, we wondered whether the immune response as quantified from the TMA cores was representative for the entire PCNSL lesions. Therefore, we assessed the intratumoral heterogeneity of the immune cell infiltrates for 26 patients of our cohort, for whom multiple tissue cores were available per tumor biopsy. Indeed, we found a marked heterogeneity in the immune response of all the lymphocyte and macrophage subsets, with non-significant to minor correlations across different multisector regions (CD3+ TILs: *R* = 0.35, *p* = 0.01; CD45ro+ TILs: *R* = 0.21, *p* = 0.11; CD68+ TAMs: *R* = 0.17, *p* = 0.19; FoxP3+ TILs: *R* = 0.25, *p* = 0.05; Figure 4A). Of note, different immune markers from the same tissue core were generally significantly correlated with each other (Figure S2). To visualize this intratumoral heterogeneity, we exemplarily plotted the spatial distribution of CD3+ cells (Figure 4B–D) across one of the rare, exceptionally large tumor resections. Overall, CD3^+^ TIL accounted for 18.5% of all cells in the entire specimen, locally ranging from below 1% to 55.7%, respectively (range 54.7%). 

## 4. Discussion

In this study, we first explored sex-specific differences in a large, population-representative cohort of patients with PCNSL using a multimodal dataset with dense clinical, MR imaging, and digital pathology annotations. Based on sex-specific cluster analyses, our derived clusters are broadly defined by the presence or absence of three key features: (i) tumor-associated immune response; (ii) MR imaging phenotype, including enhancing tumor volume (as a proxy for lesion size); and (iii) clinical presentation based on age and performance. Notably, we observed divergent prognostic constellations in female and male patients, suggesting the differential roles of MR imaging phenotypes and anti-tumor immune responses. 

The immune response differs between males and females, with stronger immune responses and higher rates of autoimmunity in females [53]. This effect is partly mediated by a differential distribution and/or activation of lymphocyte subsets [54]. While we did not observe differences in lymphocyte counts, we found an opposing prognostic role for regulatory T cells, with higher levels being associated with shorter survival in females but longer survival in males. This is of interest, since regulatory T cells were previously shown to be modulated by estrogen, which is particularly relevant during pregnancy, where they promote tolerance toward the fetus [55,56]. Our finding of a divergent role for T regulatory cells in a cancer cohort seems particularly relevant, as it may point towards differences in the response to immunotherapy which are being increasingly uncovered [57]. Overall, those subgroups of PCNSL patients with enhanced tumor-related immune responses (including TIL and TAM subsets) showed a significantly worse overall survival, particularly in female patients. This contrasts with multiple other cancer types such as non-small cell lung cancer, colorectal cancer, or melanoma [58,59,60], and prompted us to further explore the representativity of assessing a tumor’s immune response based on small, randomly taken samples such as stereotactic biopsies or tissue microarrays. Indeed, when analyzing multiple regions across the same tumor, we found significant differences between immune cold and hot areas, which puts our findings in a broader biological perspective.

While immune response seemed to be more relevant in female patients, MR imaging-derived phenotypes emerged as prognostically significant in both female and male patients, albeit in different contexts. In females, a frontal location was associated with an improved outcome; a finding that seems little surprising, given that a frontal tumor location is a favorable predictor also in other types of brain cancers such as diffuse glioma. There it is explained by better resectability and/or more favorable underlying tumor biology [61,62,63,64]. Of note, our frontally located PCNSLs were not more likely to undergo more extensive resections as compared with the stereotactic biopsies. Regarding male patients, lesions in deep locations—as defined by the involvement of the corpus callosum, periventricular regions, basal ganglia, thalamus, brain stem and cerebellum—seemed to cluster in younger patients. In this patient subset, a deep location partly diminished the positive prognostic effect of young age, which further resulted in no net survival difference as compared with the remaining male cluster of older patients with larger tumors and stronger immune responses. Of note, we observed a preferential occurrence of multifocal disease in male patients, which to our knowledge has not been previously observed and needs to be confirmed in independent data sets. Beyond tumor location, we found enhancing tumor volume to be of prognostic significance, which nicely supports the results of recent studies [25,65]. However, while those did not assess female and male patients separately, we found this association exclusively in male patients. Taken together, our MR imaging-based differences between male and female patients are not only of relevance for diagnostic assessments but might also impact the longitudinal monitoring. 

The strength of our study is that it brings together a truly multimodal set of parameters in a relatively large cohort of patients with an exceedingly rare disease such as PCNSL. Still, the sample size is an issue for detailed subgroup analyses, which prevented us from an internal cross-validation of cluster analyses or cox regression models. Additionally, due to a multiple testing problem, our analyses are primarily exploratory in nature and for hypothesis generation. Therefore, one of the most important next steps will be to further extend this cohort and validate our observations in external PCNSL cohorts. In that sense, our dataset represents one of the first comprehensive clinical/imaging/pathology datasets, so far, that serves as a starting and reference point for future studies in the field.

## 5. Conclusions

We report on a first exploratory sex-specific analysis of PCNSL using advanced cluster and survival analyses. We find tumor-associated immune response to be particularly relevant in female patients and to deviate from the patterns observed in males, whereas MR imaging-based enhancing tumor volume seems a predictor of outcomes in male patients. These results provide further evidence for sex-specific differences in brain cancer types and support the broader applications of such analyses in the field of neurooncology.

## Figures and Tables

**Table 1:** Comparisons of the parameters between the female and male patients. Numerical data is given as (mean ± standard deviation); ordinal data is given as (median (interquartile range)); nominal data is given as (absolute frequency (relative frequency)). *p*-values were calculated with *t*-tests for numerical variables, Mann–Whitney U tests for ordinal variables, and Fisher’s exact test for nominal variables. TIL: tumor-infiltrating lymphocytes. TAM: tumor-associated macrophages. HD-Methotrexate: High-dose Methotrexate (dose ≥ 3 g/m^2^).

Variable Type	Variable	Female	Male	*p*-value
Clinical prognostic/therapeutic (all as part of first line)	Age	64.6 ± 13.2 yrs	61.7 ± 14.6 yrs	0.39
	ECOG score	1 (IQR 0–2)	1 (IQR 1–2)	0.5
	Immunodeficiency	3 (7.3%)	4 (12.1%)	0.69
	Combined radio-chemotherapy	15 (36.6%)	6 (18.2%)	0.12
	Chemotherapy only	18 (43.9%)	21 (63.6%)	0.11
	Radiotherapy only	5 (12.2%)	3 (9.1%)	0.73
	Best supportive care	3 (7.3%)	3 (9.1%)	1
	Methotrexate-Tx	29 (70.7%)	27 (81.8%)	0.29
	HD-Methotrexate-Tx	27 (65.9%)	23 (69.7%)	0.81
	Rituximab-Tx	13 (31.7%)	3 (9.1%)	0.02
	Poly-chemotherapy	15 (36.6%)	10 (30.3%)	0.63
MR imaging	Enhancing volume	20.7 ± 23.5 cm^3^	18.5 ± 13.9 cm^3^	0.65
	Necrotic volume	0.4 ± 2.1 cm^3^	0.5 ± 1.8 cm^3^	0.8
	Edema volume	101.4 ± 82.3 cm^3^	92.0 ± 66.0 cm^3^	0.6
	Left location	47.0 ± 40.7%	44.0 ± 40.7%	0.75
	Frontal location	18.4 ± 27.1%	25.1 ± 31.4%	0.34
	Temporal location	11.7 ± 22.6%	9.3 ±21.9%	0.65
	Parietal location	7.0 ± 11.2%	5.5 ± 17.3%	0.66
	Occipital location	3.1 ± 9.6%	2.2 ± 6.5%	0.64
	Deep location	59.7 ± 29.3%	57.9 ± 34.5%	0.81
	Number of foci	1 (IQR 1–2)	2 (IQR 1-3)	<0.01
Digital pathology	CD3^+^ TIL	14.6 ± 12.4%	16.0 ± 19.1%	0.7
	CD45ro^+^ TIL	40.2 ± 21.4%	35.0 ± 23.9%	0.34
	CD68^+^ TAM	19.6 ± 12.4%	17.2 ± 12.3%	0.41
	FoxP3^+^ TIL	0.9 ± 1.5%	1.0 ± 1.5%	0.73

**Table 2:** Cox regression modelling performed separately for female and male patients. Hazard ratios for numerical variables are given as per percentage point increase (FoxP3, frontal location) and per cm^3^ increase (enhancing volume).

**Female patients**			
**Variable**	**HR**	**95% CI**	***p***
Age > 60 years	4.37	1.8–10.6	0.001
FoxP3 TIL	1.65	1.04–2.63	0.035
**Male patients**			
**Variable**	**HR**	**95% CI**	***p***
ECOG > 1	4.62	1.48–14.4	0.008
Enhancing volume	1.05	1.01–1.09	0.006
Frontal location	0.986	0.97–1	0.091
FoxP3 TIL	0.697	0.497–0.978	0.037

## Supplementary Materials

The following are available online at https://www.mdpi.com/2072-6694/12/6/1593/s1: Figure S1: Clustering results for the whole patient cohort, Figure S2: Distribution and correlations of different immunohistochemical markers on the same TMA tissue core, Table S1: Cluster comparisons, Table S2: Split-points and Survival for dichotomized variables obtained by recursive partitioning analysis.
