# Supplementary material for: Sex-Specific Differences in Primary CNS Lymphoma
Source: Cancers (Basel). 2020 Jun 16;12(6):1593. doi: 10.3390/cancers12061593 (PMC7352658; doi:10.3390/cancers12061593)
Supplement: Supplementary file 1 [file cancers-12-01593-s001.zip › cancers-824244-supple-conver/cancers-824244-supple-conver.pdf]

Supplementary Materials: Sex-Specific Differences in Primary CNS Lymphoma

Thomas Roetzer, Julia Furtner, Johanna Gesperger, Lukas Seebrecht, Dave Bandke, Martina Brada, Tanisa Brandner-Kokalj, Astrid Grams, Johannes Haybaeck, Melitta Kitzwoegerer, Stefan L. Leber, Franz Marhold, Patrizia Moser, Camillo Sherif, Johannes Trenkler, Julia Unterluggauer, Serge Weis, Franz Wuertz, Johannes A. Hainfellner, Georg Langs, Karl-Heinz Nenning and Adelheid Woehrer

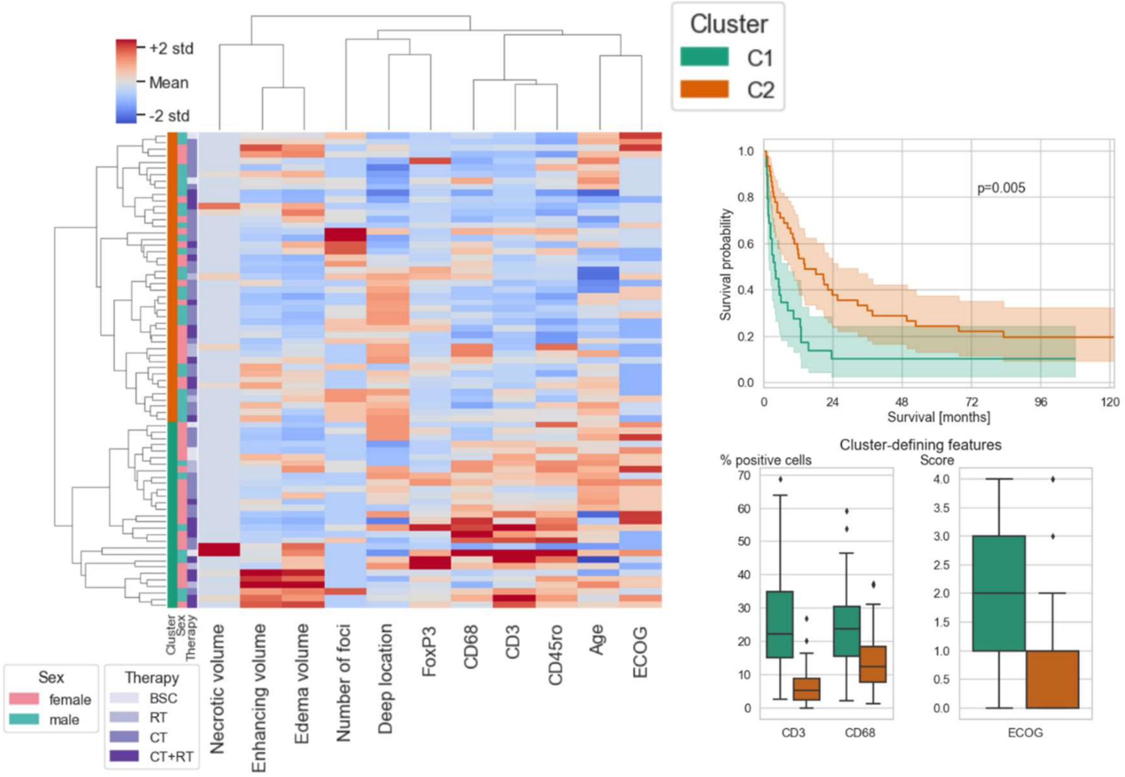

Figure S1. Clustering results for the whole patient cohort.

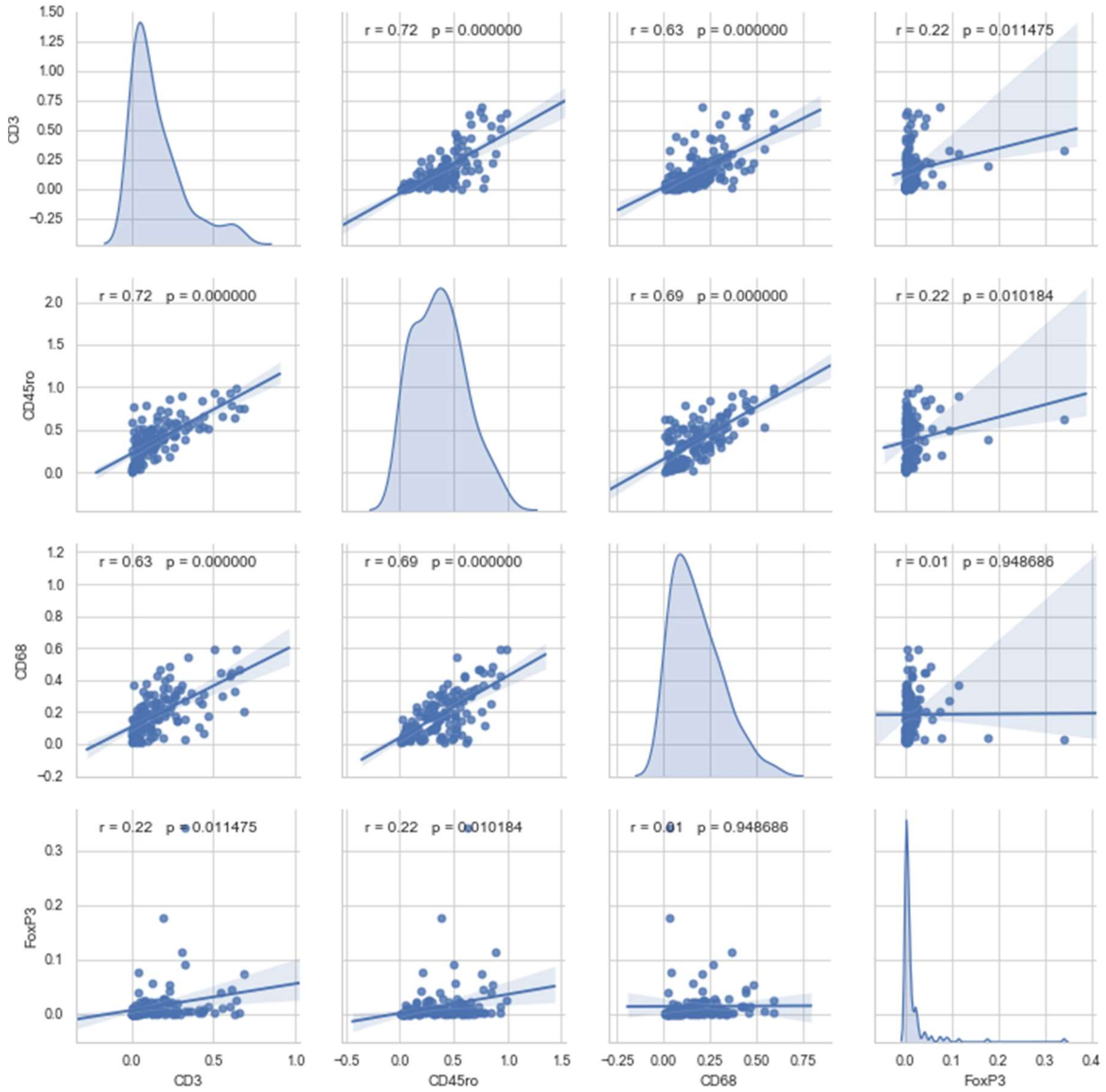

Figure S2. Distribution and correlations of different immunohistochemical markers on the same tissue microarrays (TMA) tissue core.

Table S1. Cluster comparisons. P-values were calculated with ANOVA (continuous variables) or Kruskal–Wallis *H*-tests (ordinal variables).

**Female Patients**				
	**fC1**	**fC2**	**fC3**	***p*-Value**
Age	53.5 ±10.7yrs	68.7 ±11.9yrs	68.6 ±11.6yrs	<0.01
ECOG	0 (0-1)	2 (2-2)	2 (0-3)	<0.01
Enhancing volume	10.6 ±8.2cm³	64.9 ±22.0cm³	12.0 ±9.2cm³	<0.01
Edema volume	77.1 ±68.7cm³	233.1 ±56.8cm³	73.0 ±49.8cm³	<0.01
Necrotic volume	1.3 ±4.0cm³	0.1 ±0.2cm³	0.1 ±0.3cm³	<0.01
Deep location	54.3 ±26.2%	45.3 ±14.2%	66.7 ±31.8%	<0.01

Number of foci	2 (1-3)	1 (1-1)	1 (1-1)	<0.01
CD3	4.3 ±3.4%	18.1 ±14.5%	18.4 ±11.7%	<0.01
CD45ro	17.1 ±11.3%	38.7 ±17.7%	51.7 ±16.6%	<0.01
FoxP3	0.4 ±0.5%	0.7 ±0.5%	1.1 ±2.0%	<0.01
CD68	11.6 ±7.6%	11.8 ±4.7%	25.8 ±12.4%	<0.01

**Male patients**	**mC1**	**mC2**	***p*-value**
Age	47.9 ± 13.3yrs	67.4 ± 9.5yrs	<0.01
ECOG	1 (1–2)	1 (1–1)	<0.01
Enhancing volume	4.1 ± 2.7 cm^3	24.4 ± 12.9 cm^3	<0.01
Edema volume	20.7 ± 12.3 cm^3	113.0 ± 56.8 cm^3	<0.01
Necrotic volume	0.2 ± 0.5 cm^3	0.3 ± 0.8 cm^3	<0.01
Deep location	80.8 ± 32.0%	49.4 ± 30.8%	<0.01
Number of foci	2 (1–2)	2 (1–3)	<0.01
CD3	4.3 ± 3.3%	13.7 ± 12.8%	<0.01
CD45ro	16.6 ± 9.7%	35.0 ± 18.8%	<0.01
FoxP3	0.9 ± 0.9%	0.5 ± 0.6%	<0.01
CD68	12.5 ± 7.4%	15.5 ± 9.1%	<0.01

**All patients**	**C1**	**C2**	***p*-value**
Age	60.8 ± 13.2 yrs	67.1 ± 14.2 yrs	0.06
ECOG	1 (0–1)	2 (1–3)	<0.01
Enhancing volume	17.0 ± 13.9 cm^3	24.0 ± 25.9 cm^3	0.14
Edema volume	82.7 ± 58.1 cm^3	119.7 ± 92.3 cm^3	0.04
Necrotic volume	0.2 ± 0.6 cm^3	0.9 ± 3.0 cm^3	0.16
Deep location	60.0 ± 31.7%	57.1 ± 31.6%	0.71
Number of foci	2 (1–3)	1 (1–1)	<0.01
CD3	7.0 ± 6.0%	28.0 ± 17.6%	<0.01
CD45ro	27.1 ± 16.9%	54.6 ± 20.3%	<0.01
FoxP3	0.6 ± 0.8%	1.5 ± 2.1%	0.01

CD68	14.2 ± 9.1%	25.2 ± 13.9%	<0.01

Table S2. Split-points and survival for dichotomized variables obtained by RPA. mOS: median overall survival.

	**Female Patients**				**Male Patients**				**All Patients**			
	**Groups**	***n***	**mOS**	***p***	**Groups**	***n***	**mOS**	***p***	**Groups**	***n***	**mOS**	***p***
Age	≤64 years	12	not reached	<0.0001	≤59.5 years	8	not reached	0.00038	≤59.5 years	18	not reached	<0.0001
	>64 years	19	4.21 months		>59.5 years	17	5.59 months		>59.5 years	38	5.51 months	
ECOG	≤1.5	20	12.5 months	0.024	≤1.5	18	17.4 months	0.021	≤1.5	38	13.5 months	0.0012
	>1.5	11	4.21 months		>1.5	7	4.7 months		>1.5	18	4.45 months	
Enhancing volume	≤16.3 cm³	17	7.13 months	0.23	≤5.54 cm³	7	37.7 months	0.086	≤1.99 cm³	9	82.9 months	0.058
	>16.3 cm³	14	16.8 months		>5.54 cm³	18	6.92 months		>1.99 cm³	47	10.1 months	
Necrotic volume	≤0 cm³	26	11.3 months	0.44	≤0 cm³	22	13 months	0.31	≤0.05 cm³	49	11.9 months	0.81
	>0 cm³	5	9.21 months		>0 cm³	3	1.41 months		>0.05 cm³	7	9.21 months	
Edema volume	≤55.3 cm³	13	13 months	0.11	≤23.9 cm³	7	37.7 months	0.13	≤29 cm³	13	37.7 months	0.12
	>55.3 cm³	18	9.63 months		>23.9 cm³	18	9.95 months		>29 cm³	43	10.5 months	
Left location	≤49.5%	14	9.63 months	0.32	≤58.5%	16	5.44 months	0.071	≤50.5%	29	5.88 months	0.042
	>49.5%	17	13 months		>58.5%	9	25.5 months		>50.5%	27	15.4 months	
Cerebellar location	≤0%	27	10.5 months	0.36	≤2%	18	14.1 months	0.31	≤22%	49	12 months	0.45
	>0%	4	4.21 months		>2%	7	5.59 months		>22%	7	5.59 months	
Frontal location	≤2.5%	17	7.13 months	0.046	≤13%	13	11.6 months	0.16	≤2.5%	30	8.73 months	0.015
	>2.5%	14	35.3 months		>13%	12	31.9 months		>2.5%	26	33.9 months	
Temporal location	≤7.5%	23	12 months	0.29	≤0.5%	16	12.8 months	0.63	≤7.5%	47	12 months	0.26
	>7.5%	8	7.55 months		>0.5%	9	11.9 months		>7.5%	9	9.21 months	
Parietal location	≤6%	19	9.21 months	0.37	≤1%	14	14.5 months	0.38	≤1%	29	13 months	0.53
	>6%	12	11.7 months		>1%	11	11.9 months		>1%	27	10.5 months	
Occipital location	≤0%	25	10.5 months	0.57	≤0.5%	18	9.95 months	0.87	≤1.5%	46	11.1 months	0.46
	>0%	6	11.5 months		>0.5%	7	20.7 months		>1.5%	10	12.4 months	
Deep location	≤30%	7	9.21 months	0.1	≤85.5%	15	14.1 months	0.2	≤88.5%	42	13 months	0.086
	>30%	24	11.8 months		>85.5%	10	6.92 months		>88.5%	14	6.36 months	
Number of foci	≤1.5	18	8.6 months	0.29	≤2.5	17	8.25 months	0.43	≤3.5	49	10.5 months	0.22
	>1.5	13	12 months		>2.5	8	16.3 months		>3.5	7	11.9 months	
CD3	≤3.37%	7	NA months	0.039	≤16.6%	18	17.4 months	0.29	≤15.7%	39	14 months	0.058
	>3.37%	24	9.63 months		>16.6%	7	5.59 months		>15.7%	17	5.59 months	

CD45ro	≤33.6%	17	18.2 months	0.027	≤18%	7	37.7 months	0.25	≤7.14%	8	67.6 months	0.025
	>33.6%	14	5.31 months		>18%	18	9.95 months		>7.14%	48	9.63 months	
CD68	≤19.5%	19	12.9 months	0.21	≤20.3%	18	13 months	0.31	≤27.2%	49	12 months	0.11
	>19.5%	12	4.87 months		>20.3%	7	8.25 months		>27.2%	7	2.86 months	
FoxP3	≤0.141%	7	not reached	0.049	≤1.01%	18	11.8 months	0.16	≤0.141 %	15	37.7 months	0.15
	>0.141%	24	9.63 months		>1.01%	7	49.6 months		>0.141 %	41	9.21 months	

© 2020 by the authors. Licensee MDPI, Basel, Switzerland. This article is an open access article distributed under the terms and conditions of the Creative Commons Attribution (CC BY) license (http://creativecommons.org/licenses/by/4.0/).
